# Micronutrient Deficiency and Its Potential Role in Delirium Onset in Older Adults: A Systematic Review

**DOI:** 10.1007/s12603-023-1976-z

**Published:** 2023-09-18

**Authors:** Chiara Ceolin, M.V. Papa, M. De Rui, M. Devita, G. Sergi, A. Coin

**Affiliations:** 1 Department of Medicine (DIMED), Geriatrics Division, University of Padua, via Giustiniani 2, 35128, Padua, Italy; 2 Department of General Psychology (DPG), University of Padua, via Giustiniani 2, 35128, Padua, Italy

**Keywords:** Micronutrients, delirium, older adults, vitamin D, hospitalization

## Abstract

**Background and Objectives:**

One of the pathogenetic hypotheses of delirium is the “neuroinflammatory theory” with consequent neurotoxicity of brain connectivity networks. Micronutrients may play a significant role in the prevention of neuroinflammation. This systematic review addresses the role of micronutrients in the development of delirium in older populations.

**Methods:**

The EBSCO, Cochrane, PubMed, and Web of Science databases were searched for articles on delirium and micronutrients. The methodological quality of the studies included in the review was evaluated with the Newcastle-Ottawa Scales for observational studies and for case-control studies.

**Results:**

1326 papers were identified from the searches, 7 of which met the inclusion criteria (see section 2.3). All the papers included were written in English. Delirium was predominantly secondary to post-operative dysfunction or acute medical conditions. By altering the production of neurotransmitters resulting in an imbalance, and by reducing their immunomodulatory role with a consequent increase in inflammatory oxidative stress, micronutrient deficiency seems to be associated with an increased incidence of delirium.

**Conclusions:**

This review supports the existence of an association between micronutrient deficiency (i.e. cobalamin, thiamine, and vitamin D) and an increased incidence of delirium, with a greater prevalence in hospitalized patients.

## Background

Delirium is an acute neuropsychiatric disorder encompassing disturbances in attention and cognition (1). It is the most common complication in hospitalized patients aged 65 years and over, and affects more than 2.6 million older adults each year in the United States. It is present in 8% to 17% of older patients in emergency departments, and in up to 40% of nursing home residents (1). The highest prevalence have been found in patients undergoing cardiac surgery, neurosurgery, traumatology, and radiotherapy (36% to 41%) (2). Data from the nationwide point prevalence study “Delirium Day” carried out in both acute and rehabilitation wards in Italian hospitals show a prevalence of around 23% (3).

Delirium is associated with adverse outcomes that include mortality, re-hospitalization, and institutionalization (1). The cost of health services related to delirium and its complications is estimated to be around $164 billion per year. New target interventions are therefore needed to prevent delirium and reduce its associated complications and costs (1).

One of the etiopathogenetic hypotheses of delirium is the «neuroinflammatory theory» (4). Malnutrition, i.e. a deficiency or excess of nutrients, may play a crucial role in cerebral homeostasis since nutrients are essential molecules for cellular remodeling and metabolism, and the brain is an organ with high metabolic activity and high nutritional requirements. Nutritional excesses or deficiencies could trigger a cascade of cellular events that end in cognitive changes consistent with delirium (5). In the geriatric setting, conditions such as poor nutritional status and dehydration may exacerbate or increase the risk of delirium as a secondary outcome in both cognitively compromised and normal patients. Consistent with this, delirium rates in malnourished older patients are reported to be as high as about 75% (5, 6).

Given these premises, we hypothesize that micronutrient deficiency plays a role in the onset of neuroinflammation, malnutrition, and consequently delirium. In carrying out this systematic review we attempted to identify all the available literature examining nutrient deficiency associated with delirium, and to critically appraise these studies in the context of current hypotheses of delirium pathophysiology. The ultimate aim was to revise the potential role of micronutrient deficiency in the development of delirium in older people, and, if appropriate, put forward a rational basis for future studies.

## Methods

### Systematic review tool

This review adheres to PRISMA (http://www.prismastatement.org/) and Meta-analysis of Observational Studies in Epidemiology guidelines (7).

### Identification of the studies

The EBSCO, Cochrane, PubMed, and Web of Science databases were searched for the terms “elderly”, “old”, “aged”, “advanced age”, “senior”, “geriatric”, “nutrients”, “vitamin”, “nutrition”, and “delirium” from any date to January 2023. References cited in the selected papers were examined to identify any other potential articles. After selection of papers by a first reviewer (M.DR), the whole process was repeated and confirmed by a second reviewer (C.C) to ensure validity of inclusion. Differences of opinion were discussed until consensus on inclusion or exclusion was reached with a third reviewer (M.V.P). The methodological quality of each article selected for inclusion in the review was assessed by two reviewers (M.V.P, C.C) using the Newcastle-Ottawa Scales for observational studies and for case-control studies. The data collected from each article included the study design, study country, participant characteristics, micronutrient blood levels, the methods used to measure delirium, potential confounding factors, and the findings of each study.

### Selection criteria

Inclusion criteria were: (1) studies investigating the relationship between nutrient deficiency, assessed by blood tests, and delirium; (2) studies in which delirium was assessed with a validated tool (either clinical investigation/impressions or standardized tests); (3) population age ≥65 years; (4) published studies on the topic of interest using any study methodology; and (4) availability of full text of the original research paper.

Exclusion criteria were: (1) case reports, abstracts, letters, and editorials; (2) studies not written in English; (3) animal model studies; (4) articles focusing on the therapeutic use of micronutrient supplementation to treat delirium.

### Data extraction

Titles and abstracts of selected articles were screened for relevance. The following data were extracted: (1) study design; (2) objectives; (3) incidence or prevalence of delirium; (4) delirium assessment; (5) sample size; (6) median/mean age of participants; (7) number of controls and cases; (8) assessment of variables/confounders; (9) outcome(s); (10) effects of variables/ confounders; and (11) study limitations.

## Results

**Figure 1:**
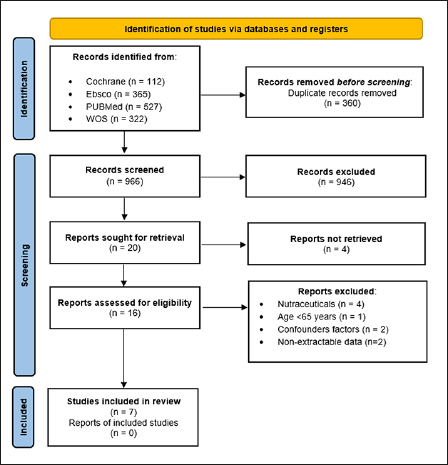
PRISMA flow diagram of the process of (and reasons for) including and excluding studies

A total of 1326 studies were identified from the database searches, of which 360 duplicates were excluded. After reviewing the titles and abstracts 946 records were discarded leaving 20 papers, the full manuscripts of which were examined in detail. A further 13 papers were discarded as ineligible, leaving 7 studies for full appraisal (Figure 1). NOS scores of the included articles are presented in Supplementary Tables 1 and 2. After evaluation by two researchers, the studies received an average NOS score of 8.0, indicative of high quality.

### Study characteristics

Of the 7 papers retained, 4 were retrospective studies, 2 prospective studies, and 1 case-control study. All dealt with micronutrients and delirium, and met the inclusion criteria of our review. One dealt with deficiencies in vitamin B12, two with thiamine deficiency, and four with vitamin D deficiency. All the articles were published between 1994 and 2021. Three of them focused on surgery populations (two cardiac, one orthopedic), three on geriatric inpatients, and one on a community-based cohort of adults. A total of 189782 participants took part in the studies. Delirium was assessed by the following means: the Diagnostic and Statistical Manual of Mental Disorders (3th and 5th editions) criteria (8, 9); the Confusion Assessment Method (10); patients' medical records; the International Classification of Diseases (10th edition) codes for delirium (11); one study used the Intensive Care Delirium Screening Checklist (12).

The studies were carried out in Canada (13), Turkey (14, 15), South Korea (16), the UK (17), Ireland (18), and Germany (19).

The individual nutrients are discussed briefly below. Table 1 lists all the selected studies demonstrating an association – whether positive or negative – between delirium and deficiency in a specific nutrient.

**Table 1:** Identified studies demonstrating associations between delirium and nutrient deficiencies

First author (year)	Type of article / study design	Country	Age mean (sd) or median (range)	Total population with micronutrient deficiencies; Sex: M/F	Population with delirium	Blood levels of micronutrients in patients with delirium	No. and type of micronutrient considered	Cut-off level used for micronutrient deficiency	Delirium diagnostic method	Confounding factor	Findings
Chouët (2020)	Unicentric case-control study	Canada	84.8 (5.7)	162	47	250HD <50 nmol/L	250HD, nmol/L	<50 nmol/L	Confusion Assessment Method.	Use of psychoactive drugs	Association between hypovitaminosis D (250HD <50 nmol/L) and delirium.
Lim (2021)	Retrospective study	South Korea	81.3 (7.2)	702; 172/530	162	250HD <20 ng/mL	25(OH)D, ng/mL	<20 ng/mL	Diagnostic and Statistical Manual of Mental Disorders, fifth edition	Hip fracture	Association between preoperative vitamin D deficiency (250HD <20 ng/mL) in hip fracture patients and onset of delirium and pneumonia.
Pilling (2021)	Prospective cohort analysis	UK	70.9 (5.71)	188806	250HD 25–50 nmol/L: 1521 with delirium; 250HD <25 nmol/L: 629 with delirium	250HD 25–50 nmol/L; 250HD <25 nmol/L	250HD	<25 nmol/L	ICD-10codeF05	NONE	Association between lower vitamin D levels and onset of hospital-diagnosed delirium in both the sufficient and deficient groups.
Turner (2020)	Retrospective study	Turkey	69.7 (7.4)	138; 16/122	48	250HD <25nmol/L.	250HD	<25 nmol/L.	The Confusion Assessment Method for the Intensive Care Unit(CAM-ICU)	Cardiac Surgery	Vitamin D deficiency exacerbates delirium after coronary artery bypass surgery with cardiopulmonary bypass.
0'Keeffe(1994)	Observational study	Ireland	83 (71–89)	6; 2/4	5	N.R.	Thiamine	Thiamine pyrophosphate effect (TPPE) greater than 25%.	Diagnosis according to DSM III	NONE	Thiamine deficiency may have been a contributory factor in the development of delirium
Pourhassan(2018)	Cross-sectional retrospective study	Germany	82.1 (7.1)	N.R.	20	Mean 54.2ng/mL	Thiamine	Blood vitamin Bl levels <20 ng/mL (59.30 nmol/L)	Diagnoses given from patients' medical records.	NONE	Mean thiamine blood levels were lower in patients with delirium (p=0.002).
Seviik (2015)	Retrospective study	Turkey	70.4 (3.8)	100; 66/34	42	160(134.2-176) ng/L	Vitamin B12	Blood vitamin B12 levels <191 ng/L	Intensive Care Delirium Screening Checklist	Coronary artery bypass graft surgery	Patients with blood vitamin B12 levels <191 ng/L had a higher incidence of delirium (42% vs 26%; p=0.017) and higher delirium severity scores (16.5±2.9 vs 15.03±2.48; p=0.034); cobalamin levels were significantly lower in patients with delirium (p=0.004).

### Cobalamin deficiency

One retrospective study was conducted on 200 patients (100 with cobalamin deficiency, mean age 71 years, and 100 controls) undergoing coronary artery bypass grafting surgery (15). Given the prevalence of neuropsychiatric disorders associated with vitamin B12 (cobalamin) deficiency, the aim was to investigate the relationship between deficiency in this micronutrient and the onset of post-cardiac surgery delirium. The authors found that cobalamin deficiency (<191 ng/L) was associated with a significantly higher incidence of delirium (42% vs 26%; p=0.017) and higher delirium severity scores (16.5±2.9 vs 15.03±2.48; p=0.034). Furthermore, cobalamin levels were significantly lower in patients with delirium than in patients without delirium (p=0.004), and delirium severity scores showed a moderate correlation with cobalamin levels (p=0.024). Logistic regression analysis showed that cobalamin deficiency was independently associated with post-operative delirium (OR 1.93, 95% CI: 1.03-3.6, p=0.038). The results of this study may have been influenced by the coronary artery bypass graft procedure as a possible confounding factor, as the authors themselves reported.

### Thiamine deficiency

In a cross-sectional retrospective study, Pourhassan et al. (19) investigated the association between blood thiamine (B1) levels and functional status, with reference also to the onset of intra-hospital delirium. The study was conducted on 233 patients (mean age 82.1±7.1) who were consecutively hospitalized in a geriatric acute care ward. Blood vitamin B1 levels of <20 ng/mL (59.30 nmol/L) were considered a deficiency. The authors reported lower mean thiamine blood levels in patients with delirium than in controls (p=0.002).

In an observational study, O'Keeffe et al. (18) examined the possible clinical manifestations of thiamine deficiency (defined as a thiamine pyrophosphate effect (TPPE) of >25%) in 36 consecutive community-dwelling older patients admitted to an acute geriatric unit. They found marginal thiamine deficiency (TPPE 15–24%) in 11 (31%) patients, and thiamine deficiency in 6 (17%). Delirium during hospital stay occurred in 6 (32%) patients with normal thiamine status, and in 13 (76%) with thiamine deficiency (p <0.025).

### Vitamin D deficiency

Five studies (13, 14, 16, 17) found a correlation between low vitamin D values measured at baseline and delirium.

Chouet et al. (13) carried out a study on 240 geriatric inpatients aged 70 years and older, 25% of whom with delirium, and reported an association between the onset of intra-hospital delirium and hypovitaminosis D, defined as blood 25-hydroxyvitamin D (25OHD) levels <50 nmol/L (OR=2.37, 95% CI: 1.07–5.25).

In the presence of vitamin D deficiency, delirium onset during hospital stay increased in hip fracture (OR 1.52, 95% CI: 1.01–2.31) and cardiac surgical patients >65 years (14, 16), and in community-based older adults (OR 2.49, 95% CI: 2.24–2.76) (17). Among the latter group, there was also a higher risk of delirium onset in the 14 day follow-up after surgery in those with insufficient 25OHD levels (OR 1.38, 95% CI: 1.28–1.49).

## Discussion

This is a comprehensive systematic review. Although the settings and delirium assessments differed between the studies, the evidence points to a possible association between micronutrient deficiency and the onset of delirium in older patients. Delirium is a frequent disorder in the multimorbid elderly patient, especially if frail, yet simply rectifying the micronutrient deficiency would not be sufficient to correct this neurocognitive disorder. Nonetheless, early identification of a state of malnutrition constitutes a potential strategy to avert the onset of delirium.

The key point, therefore, is to screen older populations for malnutrition in addition to individual nutrient deficiencies. There may be specific cognitive domains that could benefit from the correct intake of vitamins. The clinical features of delirium cover five major domains: cognitive deficits, attention deficit disorder, circadian rhythm dysregulation disorder, emotion dysregulation disorder, and psychomotor functioning impairment, with acute symptom onset and a fluctuating course (20). The mechanisms underlying delirium have not yet been elucidated in detail, although possible etiopathological mechanisms associated with micronutrient deficiency are described below.

### B-complex vitamins

Thiamine (B1) plays a role as a cofactor in the Kreb's cycle, which generates ATP. Vitamin B1 deficiency lowers the production of: ATP, leading to an increase in dopamine, in turn triggering hallucinations; acetylcholine (Ach), impairing attention; gamma-aminobutyric acid (GABA), increasing excitability; and glutathione, causing the formation of free radicals. All of these processes facilitate the development of delirium (21, 22).

A hypothesized mechanism underlying delirium in patients with cobalamin (B12) deficiency is neurotransmitter imbalances between the monoaminergic system and cholinergic neurotransmission. Specifically, there are two ways cobalamin deficiency might contribute to cognitive deterioration. First, it leads to elevated levels of homocysteine and methylmalonic acid: while homocysteine has been shown to be neurotoxic, leading to DNA damage and oxidative stress, high methylmalonic acid levels can destabilize myelin and affect normal myelin formation (23, 24, 25). Secondly, cobalamin is essential for the functioning of the central nervous system and the proper biosynthesis of neurotransmitters, such as serotonin, epinephrine, and dopamine, so its deficiency alters the metabolism of monoamine neurotransmitters and reduces protection against inflammatory oxidative stress with a consequent increase in plasma IL-2 and cortisol levels (26, 27). However, given that few studies have investigated the association between cyanocobalamin deficiency and delirium, further research is needed to clarify this relationship.

### Vitamin D

Vitamin D is a steroidal prohormone that is essential for many of the body's organs. Its receptors can be found in almost all regions of the brain (such as the cortex, hippocampus and hypothalamus) and play a crucial role in cognition (28).

Evidence indicates that vitamin D is important for both brain development (supplementation in the early years of life is suggested) and neuroprotection against neurodegenerative diseases (29). A possible effect of vitamin D is immunomodulatory, so increasing its intake can reduce the C-reactive protein ratio, and consequently inflammation. It also plays a neuroprotective role and regulates the gene expression of several neurotransmitters in the brain, such as acetylcholine, dopamine, γ-aminobutyric acid, and serotonin, all of which may be implicated in cognitive disorders, including delirium and Alzheimer's disease, a leading cause of dementia (30). Vitamin D can upregulate the expression of vitamin D receptors, and thus has neuroprotective effects against glutamate toxicity, while reducing the production of oxidative stress (31). With regard to delirium, vitamin D could offer protection due to its pleiotropic and immunomodulatory properties, its ability to cross the blood-brain barrier and to be synthesized within the nervous system, and also the ubiquitous distribution of the vitamin receptor D (also in neurons, astrocytes and oligodendrocytes) (32).

Finally, the association between vitamin D deficiency and delirium might also be explained by a possible relationship between vitamin D deficiency and physical inactivity. In fact, higher sedentary behavior was found to be associated with vitamin D deficiency, defined as <12 ng/mL, in adults and old people in a study by Solis-Urra and colleagues (33). There is, furthermore, a close relationship between delirium and physical dysfunction (34), such that the former is now considered not only a neurocognitive but also a motor disorder.

### Limitations

The major limitations of this review are the small number of available studies on geriatric populations, and our having included only publications in English. Moreover, these studies did not include people affected by multiple micronutrient deficiencies, and there was no consistency in their definitions of deficiency. Finally, the studies we retrieved were all carried out with higher-income populations, which are not representative of the world's general population. None was carried out in low- and middle-income countries (LMIC), which may limit the applicability of our findings.

## Conclusions

The results of this review support an association between deficiencies in B-complex vitamins and vitamin D and the onset of in-hospital delirium through mechanisms attributable to an increase in neuroinflammation. Subacute peripheral inflammatory stimulation induces activation of brain parenchymal cells, the expression of proinflammatory cytokines, and activation of inflammatory mediators in the central nervous system resulting in damage and possible neuronal death with subsequent decompensation of brain connectivity networks.

There is still little evidence to support the potential association between micronutrient deficiency and delirium, although various authors have repeatedly reported this relationship in hospitalized patients. Further studies with large samples representative of the global population are needed to better determine this potential association.

## Additional information

C. Ceolin and M.V. Papa reviewed the papers. All authors contributed to drafting and reviewing the text, and all approved the final article.
