# Supplementary material for: Micronutrient Deficiency and Its Potential Role in Delirium Onset in Older Adults: A Systematic Review
Source: J Nutr Health Aging. 2023 Sep 18;27(9):785–90. doi: 10.1007/s12603-023-1976-z (PMC12275627; doi:10.1007/s12603-023-1976-z)
Supplement: Supplementary file 1 — Appendix [file mmc1.docx]

**Supplementary Table 1** Study quality assessment using the Newcastle-Ottawa scale for observational studies

| Author/year | Selection | | | | Comparability  (matched analysis) |  | Outcomes |  | NOS score |
| --- | --- | --- | --- | --- | --- | --- | --- | --- | --- |
|  | Consecutive or obviously  representative series of cases | Representativeness of exposed cohort | Ascertainment of exposure | Demonstration that outcome of interest was not present at the start of study |  | Assessment of outcome | Follow-up long enough for the outcome | Adequacy of follow-up of cohorts |  |
|  |  |  |  |  |  |  |  |  |  |
| Lim (2021) | * | * | * | * | * | * | * | * | 8 |
| Pilling (2021) | * | * | * | * | * | * | * | * | 8 |
| Tumer (2020) | - | * | * | * | * | * | * | * | 7 |
| Cahill (2017) | * | * | * | * | - | * | * | * | 7 |
| O’Keeffe (1994) | * | * | * | * | * | * | * | * | 8 |
| Pourhassan (2018) | * | * | * | * | * | * | * | * | 8 |
| Sevük (2015) | * | * | * | * | ** | * | * | * | 9 |

*NOS*: Newcastle-Ottawa quality assessment scale

**Supplementary Table 2** Study quality assessment using the Newcastle-Ottawa scale for case-control studies

| Author/year | Selection | | | | Comparability of cohorts |  | Outcomes |  | NOS score |
| --- | --- | --- | --- | --- | --- | --- | --- | --- | --- |
|  | Adequate case definition | Representativeness of cases | Selection of controls | Definition of controls |  | Ascertainment of exposure | Same method of ascertainment | Nonresponse rate |  |
|  |  |  |  |  |  |  |  |  |  |
| Chouët (2017) | * | * | * | * | ** | * | * | * | 9 |

*Each asterisk represents fulfillment of an individual criterion within the subsection.
